# Randomized trial of adjuvant chemotherapy versus control after curative resection for gastric cancer: 5-year follow-up

**DOI:** 10.1054/bjoc.2000.1472

**Published:** 2001-04

**Authors:** B Neri, G Cini, F Andreoli, B Boffi, D Francesconi, R Mazzanti, F Medi, A Mercatelli, S Romano, L Siliani, R Tarquini, R Moretti

**Affiliations:** Department of Internal Medicine and Oncological Day Hospital, University of Florence, Italy; Department of General Pathology and Experimental Oncology, University of Florence, Italy; Institute of General Surgery, University of Florence, Italy; Department of Surgery, Careggi Hospital, Florence, Italy; Department of Surgery, Civic Hospital, Viareggio, Italy; Department of Clinical Physiopathology, University of Florence, Italy

**Keywords:** adjuvant chemotherapy, epidoxorubicin, 5-fluorouracil, gastric cancer, leucovorin, randomized trial

## Abstract

Adjuvant chemotherapy of gastric cancer after curative resection is still subject to discussion. In this study 137 patients with gastric adenocarcinoma, all with positive nodes, were randomized after curative resection so that 69 received epidoxorubicin (EPI), leucovorin (LV) and 5-fluorouracil (5-FU) on days 1–3 every 3 weeks for 7 months, whereas the remaining 68 did not. After a follow-up period of 5 years, 21 of the 69 treated patients (30%) and nine controls (13%) were still alive; median survival time was 18 months for the controls and 31 months for the patients treated with adjuvant chemotherapy (*P*< 0.01). © 2001 Cancer Research Campaign http://www.bjcancer.com


*British Journal of Cancer* (2001) **84**(7), 878–880
© 2001 Cancer Research Campaign
doi: 10.1054/ bjoc.2001.1472, available online at http://www.idealibrary.com on IDEAL

http://www.bjcancer.com

Short Communication

### Randomized trial of adjuvant chemotherapy versus control after curative resection for gastric cancer: 5-year follow-up

**B Neri[1], G Cini[2], F Andreoli[3], B Boffi[4], D Francesconi[5], R Mazzanti[1], F Medi[5], A Mercatelli[4], S Romano[6], L Siliani[4], R Tarquini[1] and R Moretti[4]**

[1]Department of Internal Medicine and Oncological Day Hospital, University of Florence, Italy; [2]Department of General Pathology and Experimental Oncology, University of Florence, Italy; [3]Institute of General Surgery, University of Florence, Italy; [4]Department of Surgery, Careggi Hospital, Florence, Italy; [5]Department of Surgery, Civic Hospital, Viareggio, Italy; [6]Department of Clinical Physiopathology, University of Florence, Italy

**Summary** Adjuvant chemotherapy of gastric cancer after curative resection is still subject to discussion. In this study 137 patients with gastric adenocarcinoma, all with positive nodes, were randomized after curative resection so that 69 received epidoxorubicin (EPI), leucovorin (LV) and 5-fluorouracil (5-FU) on days 1–3 every 3 weeks for 7 months, whereas the remaining 68 did not. After a follow-up period of 5 years, 21 of the 69 treated patients (30%) and nine controls (13%) were still alive; median survival time was 18 months for the controls and 31 months for the patients treated with adjuvant chemotherapy ($P < 0.01$). © 2001 Cancer Research Campaign http://www.bjcancer.com

**Keywords**: adjuvant chemotherapy; epidoxorubicin; 5-fluorouracil; gastric cancer; leucovorin; randomized trial

Gastric cancer represents the third most common cause of cancer deaths in Italy (Decarli and La Vecchia, 1988). Following curative surgery, patients with nodal involvement have an 85–90% probability of dying within 5 years (Davis et al, 1990), with a median survival of 8 months (Alexander et al, 1997). Yet, even today, adjuvant chemotherapy of gastric cancer after curative resection remains controversial. In 1996, our group published preliminary positive survival results of a randomized trial in favour of the use of adjuvant chemotherapy versus no further treatment in resected gastric cancer patients (Neri et al, 1996).

Here we present the results of our trial update with more patients accrued and longer follow-up (5 years), that compared EPI-LV-5FU adjuvant chemotherapy versus no treatment on resected, node-positive gastric cancer patients.

### PATIENTS AND METHODS

In our earlier study (Neri et al, 1996), we presented data derived from 55 patients comprising the control group (Arm A) and 48 patients treated with the EPI-LV-5FU adjuvant chemotherapy protocol (Arm B) based on an interim analysis after 36 months of observation. In this report, we provide the complete data on all 137 patients enrolled, after a 5-year follow-up period. Patients were entered into the study by 6 centres in Italy and all had histologically confirmed gastric adenocarcinoma without clinical or radiologic evidence of distant metastases, a Karnofsky score greater than 60 and past good general health with no history of cardiac disorder or congestive heart failure. Table 1 outlines clinical characteristics of the patients and their tumour stage. All patients were aware of the investigational nature of the treatment and had given written informed consent, in line with institutional regulations. Full staging of patients was carried out before they entered into the trial. In the randomization carried out 4–6 weeks following gastric resection, patients were stratified by centre to receive either postoperative chemotherapy with Epidoxorubicin

**Table 1** Clinical characteristics of patients

| | Treatment arm | |
|---|---|---|
| | **Control (arm A)** | **Chemotherapy (arm B)** |
| Evaluable patients | 68 | 69 |
| Median age (range) | 64 (35–74) | 62 (37–73) |
| Sex | | |
| Male | 48 | 50 |
| Female | 20 | 19 |
| Site of primary tumour | | |
| Pylorus or antrum | 25 | 23 |
| Body | 31 | 29 |
| Cardia or fundus | 12 | 17 |
| T stage[a] | | |
| T1 | 1 | 2 |
| T2 | 6 | 9 |
| T3 | 33 | 31 |
| T4 | 28 | 27 |
| N stage[a] | | |
| N1 | 30 | 35 |
| N2 | 38 | 34 |
| Surgery | | |
| R-1A resection | 21 | 23 |
| R-1B resection | 37 | 37 |
| R-2 resection | 10 | 9 |
| Karnofsky score | | |
| ≥ 80 | 35 | 39 |
| < 80 | 33 | 30 |

[a]International Union Against Cancer (1987)

*Received 6 April 2000*
*Revised 31 July 2000*
*Accepted 9 August 2000*

*Correspondence to:* B Neri

**Table 2** Hazard ratio[a] and confidence limits

| Treatment | Hazard | LCL[b] | UCL[b] |
|---|---|---|---|
| Arm A | 1.96 | 1.32 | 2.92 |
| Arm B | 1.00 | – | – |

[a]Analysis for 60 months of follow-up. [b]95% Confidence limits. Arm A: controls. Arm B: treated patients.

(EPI) 75 mg/m$^2$ day 1 and Leucovorin (LV) 200 mg/m$^2$ plus 5-fluorouracil (5-FU) 450 mg/m$^2$ days 1–3 or control follow-up. Patients in both groups were evaluated at 8-week intervals during the first postoperative year, at 3-month intervals during the second and third years and at 6-month intervals in the fourth and fifth years. Treatments, evaluation of toxic effects and follow-up were carried out as reported previously (Neri et al, 1996). Postoperative 5-year survival was determined for all patients and was measured from the date of randomization to death or last follow-up.

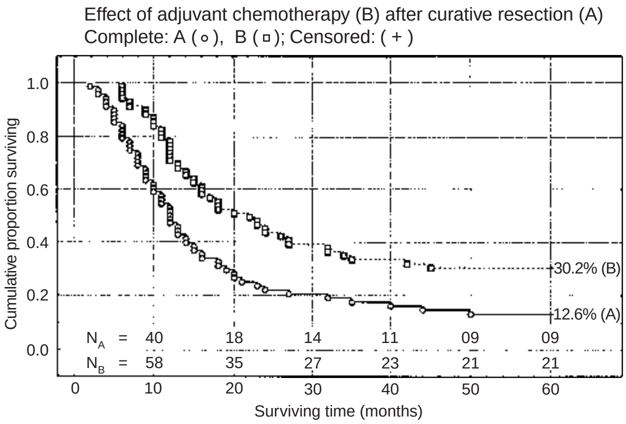

**Figure 1** Survival distribution of patients following surgery. The number of patients (risk set) is shown beneath the time axis. Arm A, controls; arm B, treated patients.

### Statistics

Life-table estimates were computed using life-table options from a univariate analysis and were compared using the log-rank test and an estimate of the hazard ratio (HR) provided with associated confidence intervals. To rule out covariates, we tested the differences in frequencies in the two patient groups (Arm A and B) by contingency table analysis (SAS Institute, 1987).

### RESULTS

This is the second and final publication on 137 randomized patients with gastric cancer after a 5-year follow-up period. A total of 402 chemotherapy cycles were recorded. 61 patients (88%) received all of the planned 7 cycles of the EPI-LV-5-FU schedule. Two patients developed severe myelosuppression and completed only 4 and 5 cycles respectively, with an attenuated dose. Three patients refused to go on with therapy after the fourth cycle and one after the fifth cycle. Two others relapsed after the third and fourth cycle and died 7 and 9 months after the onset of treatment. The total observation period extended over 5 years. The median survival time for the 68 untreated patients was 18 months (range 2–60+). The 69 treated patients had a median survival time of 31 months (7–60+), a significant increase ($P < 0.01$), and HRs calculated for the whole period of observation support these findings (Table 2). In the control group 59 out of 68 patients died because of recurrence vs 48 out of 69 in the adjuvant EPI-LV-5-FU treated group. Survival time and the proportion of patients alive by the end of 60 months of observation are reported in Figure 1.

Our multivariate analysis took into account 3 potentially confounding factors: stage, lymph node status and type of surgery. We obtained the following results: $P > 0.33$ for stage; $P > 0.43$ for lymph node status and $P > 0.75$ for surgery, leading us to conclude that treatment was the only significant prognostic factor.

Toxicity scores among patients are listed in Table 3. Myelosuppression tended to be cumulative, with lower and more prolonged nadirs after 5 cycles. Severe leucopenia affected only 5 patients. None of our patients required hospitalization for sepsis, and 10 who experienced infection (mainly pulmonary) were all manageable on an outpatient basis.

### DISCUSSION

In Western countries, postoperative gastric cancer adjuvant strategies have until now not succeeded in improving overall survival (Coombes et al, 1990; Kelsen, 1996), even though the Japanese data strongly suggest that adjuvant chemotherapy should be an integral part of the treatment of patients with gastric cancer after curative resection. In fact their data appear so convincing that, since 1982, they have abolished the control group in their studies (Nakajima and Nishi, 1989). Along with others (The

**Table 3** Grade of toxicity according to World Health Organization

| | Grade | | | | | Grade 3 or 4 toxicity | |
|---|---|---|---|---|---|---|---|
| | 0 | 1 | 2 | 3 | 4 | Incidence | Percentage |
| Emesis | 25 | 27 | 17 | – | – | – | – |
| Diarrhoea | 17 | 28 | 18 | 6 | – | 6/69 | (8.7) |
| Mucositis | 18 | 23 | 20 | 8 | – | 8/69 | (12.0) |
| Alopecia | 14 | 23 | 32 | – | – | – | – |
| Cardiac | 25 | 30 | 14 | – | – | – | – |
| Hepatic | 25 | 22 | 12 | – | – | – | – |
| Neurological | 35 | 34 | – | – | – | – | – |
| Renal | 30 | 34 | 5 | – | – | – | – |
| Anaemia | 21 | 25 | 20 | 3 | – | 3/69 | (4.3) |
| Leucopenia | 20 | 21 | 22 | 5 | 1 | 6/69 | (8.7) |
| Thrombopenia | 21 | 28 | 18 | 2 | – | 2/69 | (2.9) |

© 2001 Cancer Research Campaign 

Gastrointestinal Tumor Study Group, 1982; Michelassi et al, 1994), we considered the presence of lymph node involvement a highly unfavourable prognostic factor for gastric cancer patients, hence one requiring adjuvant treatment. The results of our study after 5 years confirm our previous findings (Neri et al, 1996) and the conclusions of a more recent meta-analysis (Earle et al 1998) that adjuvant chemotherapy produces a small survival benefit in patients with curatively resected gastric carcinoma. Those with lymph node metastases have a higher risk of recurrence and may derive more absolute benefit from the treatment. In the future, to better select patients with a greater likelihood of profiting from adjuvant chemotherapy, we intend to supplement data on lymph node involvement with an analysis of the tumour's biomolecular characteristics (Yonemura et al, 1996; Fenoglio-Preiser, 1997) based on the study of its cellular proliferation, invasion and resistance to chemotherapy. We hope that our results will be confirmed on larger samples and, if possible, improved with more active treatment schedules.

### ACKNOWLEDGEMENTS

The authors would like to acknowledge all those who have assisted us in this study and especially Dr Eda Berger. The study was partially funded by the 'Associazione Toscana Cure e Ricerche Oncologiche' and by the bank 'Monte dei Paschi di Siena'.

### REFERENCES

Alexander AR, Kelsen DP and Tepper JE (1997) Cancer of the stomach. In: DeVita VT, Hellman S and Rosemberg SA (eds), *Cancer: Principles and Practice of Oncology* pp. 1021–1054

Coombes RC, Schein PS, Chilvers CED, Wils J, Beretta G, Bliss JM, Rutten A, Amadori D, Cortes-Funes H and Villar-Grimalt A (1990) A randomized trial comparing adjuvant fluorouracil, doxorubicin and mitomycin with no treatment in operable gastric cancer. *J Clin Oncol* **8**: 1362–1368

Decarli L and La Vecchia C (1988) Cancer mortality in Italy. *Tumori* **74**: 6623–6632

Davis LD, Hoel D, Fox J and Lopez A (1990) International trends in cancer mortality in France, West Germany, Italy, Japan, England and Wales and the USA. *Lancet* **336**: 474–481

Earle CC and Maroun JA (1999) Adjuvant chemotherapy after curative resection for gastric cancer: revisiting a meta-analysis of randomized trials. *Europ J Cancer* **35**: 1059–1064

Fenoglio-Preiser CM (1997) The effect of oncogenes on the biology and prognosis of surgically resected gastric cancer. *ASCO Educational Book* pp. 275–277

International Union Against Cancer (1987). *Classification of Malignant Tumours*, Hermanek P and Sobin LH (eds). Springer: Geneva

Kelsen DP (1996) Adjuvant and neoadjuvant therapy for gastric cancer. *Semin Oncol* **23**: 379–389.

Michelassi F, Takanishi DM, Pantalone D, Hart J, Chappel R and Block GE (1994) Analysis of clinicopathologic prognostic features in patients with gastric adenocarcinoma. *Surgery* **116**: 804–810.

Nakajima T and Nishi M. (1989) Surgery and adjuvant chemotherapy for gastric cancer. *Hepatogastroenterology* **36**: 79–85.

Neri B, de Leonardis V, Romano S, Andreoli F, Pernice LM, Bruno L, Borrelli D, Valeri A, Fabbroni S, Intini C and Cini G. (1996) Adjuvant chemotherapy after gastric resection in node-positive cancer patients: a multicentre randomised study. *Brit J Cancer* **73**: 549–552

SAS Institute Inc. (1987) SAS/STAT Guide for Personal Computers, Version 6 edn. SAS Institute: Cary, NC

The Gastrointestinal Tumor Study Group (1982) Controlled trial of adjuvant chemotherapy following curative resection for gastric cancer. *Cancer* **49**: 1116–1122

Yonemura Y, Kaji M and Fushida S (1996) Correlation between over-expression of c-met gene and the progression of gastric cancer *Int J Oncol* **8**: 555–560.

 *© 2001 Cancer Research Campaign*

